# Comparison of machine learning algorithms applied to symptoms to determine infectious causes of death in children: national survey of 18,000 verbal autopsies in the Million Death Study in India

**DOI:** 10.1186/s12889-021-11829-y

**Published:** 2021-10-04

**Authors:** Susan Idicula-Thomas, Ulka Gawde, Prabhat Jha

**Affiliations:** 1 Biomedical Informatics Centre, Indian Council of Medical Research-National Institute for Research in Reproductive Health, Mumbai, 400012 India; 2 Centre for Global Health Research, St. Michael’s Hospital, Unity Health Toronto, and Dalla Lana School of Public Health, University of Toronto, Toronto, Ontario Canada

**Keywords:** Machine learning, Prediction model, Million Death Study, Verbal autopsy, Child mortality, Cause of death, Infectious disease

## Abstract

**Background:**

Machine learning (ML) algorithms have been successfully employed for prediction of outcomes in clinical research. In this study, we have explored the application of ML-based algorithms to predict cause of death (CoD) from verbal autopsy records available through the Million Death Study (MDS).

**Methods:**

From MDS, 18826 unique childhood deaths at ages 1–59 months during the time period 2004–13 were selected for generating the prediction models of which over 70% of deaths were caused by six infectious diseases (pneumonia, diarrhoeal diseases, malaria, fever of unknown origin, meningitis/encephalitis, and measles). Six popular ML-based algorithms such as support vector machine, gradient boosting modeling, C5.0, artificial neural network, k-nearest neighbor, classification and regression tree were used for building the CoD prediction models.

**Results:**

SVM algorithm was the best performer with a prediction accuracy of over 0.8. The highest accuracy was found for diarrhoeal diseases (accuracy = 0.97) and the lowest was for meningitis/encephalitis (accuracy = 0.80). The top signs/symptoms for classification of these CoDs were also extracted for each of the diseases. A combination of signs/symptoms presented by the deceased individual can effectively lead to the CoD diagnosis.

**Conclusions:**

Overall, this study affirms that verbal autopsy tools are efficient in CoD diagnosis and that automated classification parameters captured through ML could be added to verbal autopsies to improve classification of causes of death.

**Supplementary Information:**

The online version contains supplementary material available at 10.1186/s12889-021-11829-y.

## Background

The ongoing COVID-19 pandemic has sharply revealed the long standing fact that many of the deaths, especially in low-income countries are not well documented as most of the deaths occur at home and not in well-regulated hospital settings. A second reason for poor documentation of death is because unlike birth, family members are not sufficiently incentivised to register death. This gap in death records and associated data is a serious impediment in assessing disease patterns and public health needs of a country. To address this gap, the Million Death Study (MDS) was initiated in India to quantify premature mortality through verbal autopsy (VA) [1, 2] in a nationally representative sample of homes. VA uses a set of symptoms and signs captured through a structured questionnaire to assign cause of death (CoD) [3–5]. The questionnaire is administered to family or caretakers of the deceased by non-medical surveyors. Each data record is then assigned randomly to two of the several trained physicians in the team. The physicians independently assign the CoD, based on the surveyor’s report. In cases, where the CoD assignment for a record does not match for the two physicians, it is adjudicated by a third senior physician.

It would be worthwhile to study how efficiently the signs and symptoms captured by the surveyors could be used to predict CoD using supervised machine-learning (ML) algorithms. Such a study, in addition to revealing the scope for automation of VA tools, will also give insights on improvement of methodology for more accurate diagnosis at reduced cost of implementation.

Supervised ML algorithms learn from a set of input variables to predict a response variable. Many of the classification problems in biological and medical fields have been successfully solved using ML methods such as support vector machine (SVM), gradient boosting modelling (GBM), C5.0 (C5), artificial neural network (ANN), k-nearest neighbour (kNN), classification and regression tree (CART) [6, 7]. SVM and ANN algorithms have been successfully used for disease detection [8–11].

In this study, MDS dataset captured from 2004 to 2013 for ages 1–59 months has been explored for ML-based prediction of CoD for six infectious diseases viz. pneumonia, diarrhoeal diseases, malaria, meningitis/encephalitis, measles and fever of unknown origin (FOUO).

## Methods

### Population-based mortality data

The rationale, methodology, and efficacy of the MDS have been described elsewhere [12, 13]. The RHIME (Routine, Reliable, Representative and Re-sampled Household Investigation of Mortality with Medical Evaluation) form was used by trained surveyors to obtain information from family or caretakers of the deceased [14]. Each completed survey in the MDS was reviewed independently by two trained physicians, who were randomly assigned VAs through an online portal based on matching language proficiency of the physician and the language in which the VA was completed. Two independent physicians reviewed all the completed RHIME forms and assigned the underlying CoD according to the International Classification of Diseases, tenth revision (ICD-10) [15], and included a number of “keywords” in the record, which are signs and symptoms observed in the VA that support their diagnosis. The CoD was approved for records wherein the two physicians assigned the same CoD. For the remaining records, a third senior physician was referred to finalise the CoD based on the physicians keywords [2, 16]. Initial differences in coding (about 30% of records) were reconciled by both physicians, who each anonymously received the other’s keywords justifying their choice of underlying CoD. After this reconciliation stage, any outstanding differences were assigned to and adjudicated by one of 40 senior physicians (about 10% of records). The steps involved in the MDS underwent various quality assurance checks, including resampling by an independent team in 2001–2003 that yielded similar results to the original survey.

The MDS records obtained for India from 2004 to 2013 were filtered for age between 1 to 59 months and cases wherein both physicians initially agreed on the underlying CoD. These filtering criteria led to 18,826 unique records and this data was further segregated based on six infectious disease categories: pneumonia, diarrhoeal diseases, malaria, fever of unknown origin, meningitis/encephalitis, and measles (Table 1). Previous analyses by Dingra et al. and review of ICD coding by Aleksandrowicz et al. suggest ‘fever of unknown origin’ as predominantly infectious, thus we have included it in the infectious disease category [17, 18].

**Table 1:** Number of MDS 2004–13 VA records with initial physician agreement for ages 1–59 months across six infectious causes of death

Cause of death (CoD)	Disease code	ICD-10 codes	Number of cases	% cases
Pneumonia	Pneum	A37, J00-J06, J09-J18, J20-J22, J32, J36, J85, J86, P23, U04	5733	43
Diarrhoeal diseases	Diar	A00-A09	4897	37
Malaria	Mal	B50-B54	860	7
Fever of unknown origin	Fouo	R50	754	6
Meningitis/encephalitis	Men	A39, A81-A89, G00-G09	490	4
Measles	Meas	B01, B05	482	4
Total		All above codes	13,216	

These six diseases constituted ~ 70% (13,216 out of 18,826) of the total deaths across all CoDs in this age category. The remaining 30% (5610 out of 18,826) constituted the other five diseases such as tuberculosis, injury, non-communicable disease (NCD), ill defined conditions (ILDF), and communicable, perinatal and nutritional disorders (CMPND).

### Data processing

Physicians keywords for each record were aggregated for both physicians and grouped into 35 symptom categories and subcategories, selected based on their medical relevance to the six CoD included in this study. The 35 groupings as well as inclusion and exclusion terms for symptom categories and subcategories are shown in S1 Appendix. Symptom groups were coded in a binary fashion: each of the 18,826 records received a “1” if either coding physician listed keywords reflecting the symptom category, and a “0” if they did not. Four of the symptom categories (fever, breathing problems, cough, diarrhoea) also contained subcategories that were aggregated under the parent category (S1 Appendix). For example, if one of the physician keywords for a death record was “high fever,” the record was coded to reflect both the “fever” and “high fever” categories. Stata version 14.2 [19] was used for the physician keyword classification.

### ML-based algorithms for prediction of CoD

Machine learning (ML) algorithms are popularly used for predicting an outcome or dependent variable from a pool of high dimensional input variables. In this study, the outcome or dependent variable is the CoD assignment and the input variables are physician’s keywords for each record. The ML algorithms such as support vector machine (SVM), gradient boosting modelling (GBM), C5.0 (C5), artificial neural network (ANN), k-nearest neighbour (kNN), classification and regression tree (CART) were implemented using *e1071*, *rpart*, *gbm* and *caret* R packages with default parameter settings [20–23]. In case of SVM, the radial basis function (RBF) kernel was selected for transforming the input features into the high dimensional space for hyperplane differentiation of the positive and negative classes. RBF is known to be more generalized and robust as compared to the other kernel functions available for SVM [24]. The values for cost ‘C’ and ‘sigma’ were optimised for each model individually.

#### SVM

SVM, as a supervised machine learning algorithm, can be used for generating classification and regression models. For classification models, SVM algorithms plot each record of a dataset as a point in n-dimensional space, where n is the number of numerical features for each record and creates a hyperplane for the separation of two or more classes of datasets. The points closest to the hyperplane/separator are called support vectors as it holds the separating plane. The algorithm aims to generate a hyperplane that maximises the distance between the classes/datasets and simultaneously minimises the classification errors. In cases where data points are not linearly separable, SVM uses the kernel function [6, 25–29].

#### ANN

ANN algorithms function by mimicking the biological nervous system, which has many neurons connected in a layered manner. ANNs consist of an input layer that captures the features/variables of the datasets; one or more hidden layers which process the information, and an output layer that displays the outcome. Each variable can be denoted as a node and their interactions are denoted by edges. ANN can detect non-linear relationships between variables and generate predictions based on nodes, and edge weights. The advantages of ANNs are its tolerance to noise, capability of learning complex data, and classify instances into more than one output. For large neural networks, the interpretation of the algorithm may be difficult and can require high processing time [6, 25, 27, 29, 30].

#### kNN

kNN is a supervised machine learning algorithm which is conceptually simple, and non-parametric in nature. kNNs work by capturing the closest data points for the query record to a known dataset and then assign the class of query based on majority of class votes. The input features of the dataset are used to identify the closeness between the records. Here, k denotes the number of closest data points considered for the vote and hence is an important parameter for the prediction outcome. The advantages of kNNs are its easy implementation, quick learning and not prone to overfitting. The disadvantages of kNN are its sensitivity to noise, and requirement of large storage space [6, 27].

#### CART

CART is a decision tree-based algorithm in which each root node of a tree represent an input variable and leaf nodes of tree represent the output variable. A binary decision tree is generated at each step by splitting a node into two child nodes. It creates a set of logical rules, the response to which determines the split in the dataset. The advantages of CART algorithm include fast processing of data and easy interpretation of the algorithm [31–33].

#### GBM

GBM is a tree-based method that combines predictions from multiple decision trees. Each of the decision trees can be considered as weak learners which eventually are converted into strong learners by minimising the errors of the previous decision tree. The advantages of GBM include its high predictive accuracy and ability to predict multiclass data. The disadvantages of GBM include overfitting of data, sensitivity to noisy data, and requirement of high processing time [28, 34, 35].

#### C5

C5 is also a tree-based algorithm that functions by minimising the information entropy or maximising the information gain at each split. The data is split initially based on the biggest information gain and continued till it cannot be split further. The features that do not contribute to the splits are removed from the final model. While C5 algorithms are easy to implement and interpret, it requires categorical (ordinal/nominal) data as target variable and may not work well on small datasets [31, 36].

### Generation of training and test datasets

Individual prediction models were generated for each of the six infectious diseases namely pneumonia, diarrhoeal diseases, malaria, meningitis/encephalitis, measles and fever of unknown origin (FOUO). For each disease model, records belonging to the disease that is being predicted were marked as positive and the remaining records (not limited to the 6 diseases considered in the study) are marked as negative. An unbalanced dataset can be converted to balanced dataset (with equal representation from positive and negative classes) by random resampling either by oversampling the minority class or undersampling the majority class. Here, we opted for creation of balanced 2-class classifier by undersampling the majority class (negative class) to match the number of records in minority class (positive class) for each of the six disease datasets. Subsequently, for each of the models, the dataset was partitioned into training and test datasets using 80:20 random split. The robustness of each ML-based model was evaluated by performing a 10-fold cross-validation with 10 iterations on the training dataset.

The SVM prediction models were also generated to differentiate between pair of diseases with overlapping symptoms such as i) pneumonia and diarrhoeal diseases, ii) malaria and meningitis/encephalitis, and iii) malaria and FOUO. In these cases, the positive and negative classes comprised of the records of first and second disease respectively and undersampling of the majority class was used to generate a balanced classifier.

### Evaluation of prediction models

The test datasets were used to evaluate the performance of each of the selected models using the below performance metrics:

1 $$ accuracy=\frac{TP+ TN}{TP+ FN+ TN+ FP} $$

2 $$ recall/ sensitivity=\frac{TP}{TP+ FN} $$

3 $$ specificity=\frac{TN}{TN+ FP} $$

4 $$ precision=\frac{TP}{TP+ FP} $$

5 $$ F1=2\times \frac{precision\times recall}{precision+ recall} $$

*TP* (true positives) and *TN* (true negatives) denote the number of outcomes where the model correctly predicts the positive and negative class respectively. *FP* (false positives) and *FN* (false negatives) denote the number of outcomes where the model incorrectly predicts the positive and negative class respectively.

Cohen’s kappa evaluates model by measuring the agreement between predicted accuracy and observed class accuracy.

6 $$ Cohe{n}^{\prime }s\  kappa=\frac{Po- Pe}{1- Pe} $$

where *Po* is the relative observed agreement and *Pe* is the random chance of agreement [37].

### Hierarchical clustering of physicians’ keywords

The relationship/co-occurrence of symptoms reported for each disease were studied using ascendant hierarchical clustering using *hclustvar* function in *ClustOfVar* R package [38]. The number of clusters was set to six, as we were studying six diseases.

## Results

### Disease-wise distribution of records

Amongst these six diseases, deaths due to pneumonia and diarrhoeal diseases were most common, respectively reflecting 43 and 37% of the deaths in the dataset, while deaths due to measles and meningitis/encephalitis were the least common, each reflecting just under 4% of the deaths in the dataset (Table 1).

Pneumonia and diarrhoeal diseases are known to be major cause of childhood mortality in India, especially in poorer communities [39] and this is reflected in the MDS data.

### Distribution of symptoms across disease datasets

**Fig. 1:**
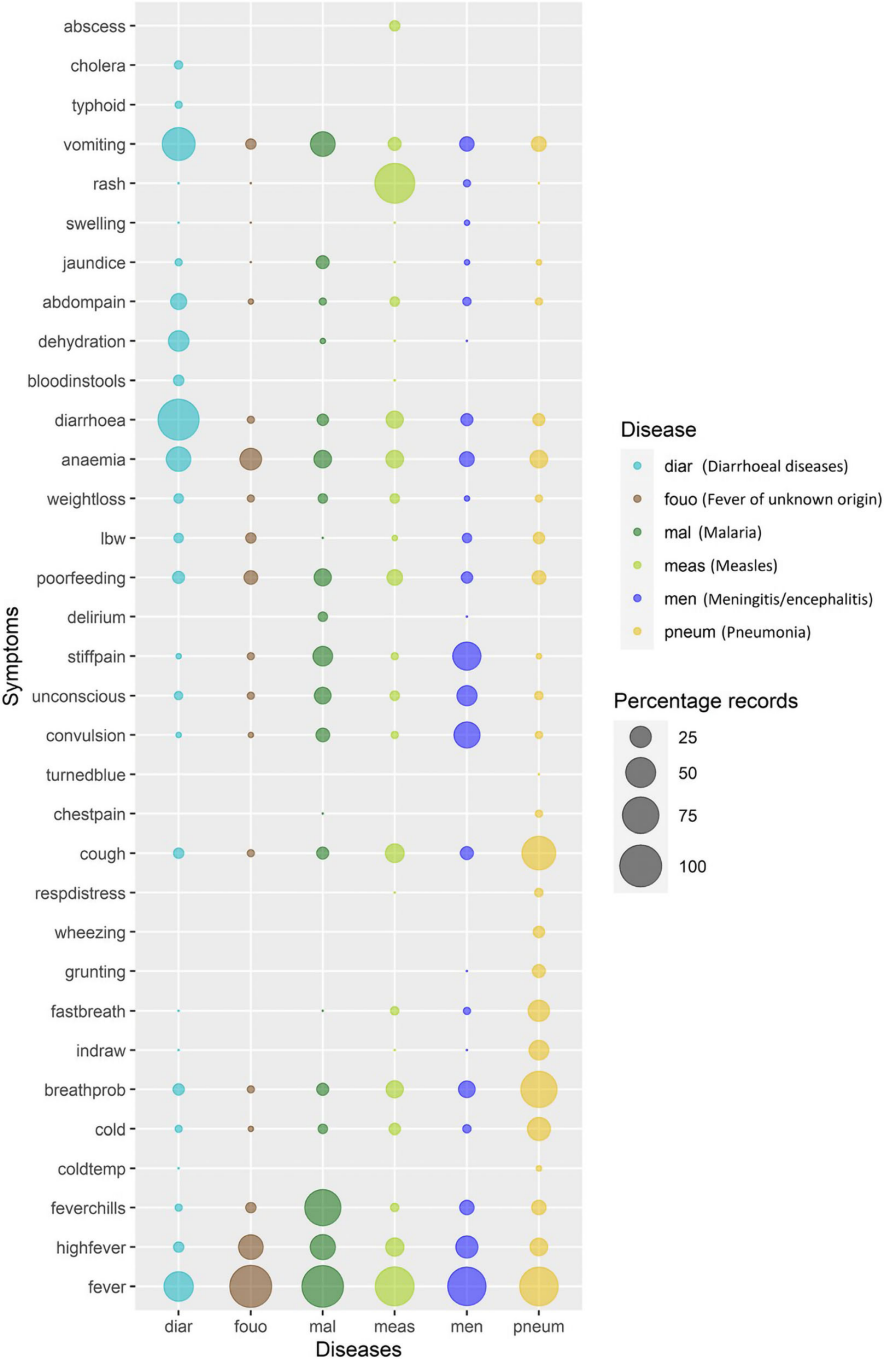
Bubble plot depicting distribution of symptoms across six infectious diseases. X-axis represents *disease* class and y-axis represents *symptoms* coded by rule-based method. The bubble size is proportional to percentage of records positive for the symptom in the disease class. The plot was generated using *ggplot2* R package [40]

In VA, physicians use the questionnaire notes of non-medical surveyors to identify keywords that eventually form the basis of CoD assignment. In this study, these keywords were converted to rule-based, non-redundant set of symptoms for ease of automation (S1 Appendix). The distribution of these symptoms across the six CoD are visualised in Fig. 1, and record counts for each of the six CoD can be found in S2 Appendix. It was observed that 17 of the 35 symptoms viz. vomiting, jaundice, abdominal pain /distention (abdompain), diarrhoea, anaemia, weight loss, low birth weight (lbw), poor feeding, stiffness/ body pain (stiffpain), unconsciousness (unconscious), convulsion, cough, breathing problems (breathprob), cold, fever chills, high fever and fever were present, with varying frequencies, in all six diseases. For e.g., vomiting and diarrhoea were frequent in diarrhoeal diseases; fever chills were present in most of malarial cases; rash was common in measles and breathing problems were observed in most of pneumonia cases. These observations were in concordance with WHO manual for disease diagnosis [41–43].

**Fig. 2:**
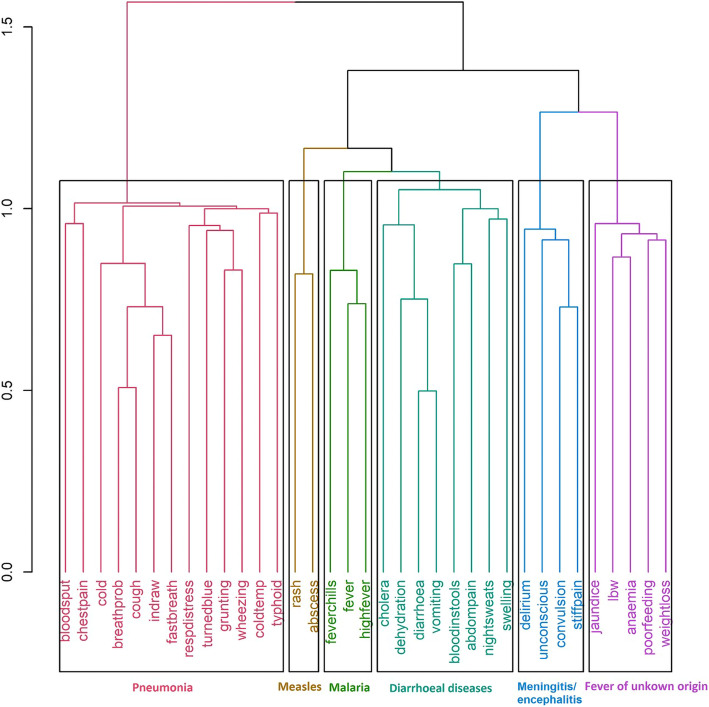
Tree-based clustering of symptoms for six clusters. The vertical axis represents distance between clusters

To evaluate if symptoms can self-cluster, based on their co-occurrence into distinct disease classes, unsupervised (without CoD annotation) hierarchical clustering was performed on 13,216 records belonging to six diseases (Fig. 2).

The clustering algorithm was forced to generate six clusters and interestingly the six clusters represented the six CoDs as can be deduced based on the nature of symptoms. Cluster 1 had symptoms such as breathing problem (breathprob), cough, cold, chest indrawing (indraw), fast breathing (fastbreath), grunting, respiratory distress (respdistress) and wheezing which are attributable to Pneumonia; Cluster 2 had rash and abscess that is characteristic of measles; Cluster 3 had fever chills, fever, high fever typical of malaria; Cluster 4 had cholera, dehydration, diarrhoea, vomiting, blood in stools, abdominal pain /distention (abdompain), night sweats and swelling that are commonly observed in diarrhoeal diseases; Cluster 5 had delirium, unconsciousness, convulsion and stiffness/body pain (stiffpain) distinctive features of meningitis/encephalitis and Cluster 5 had jaundice, low birth weight (lbw), anaemia, poor feeding and weight loss representing fever of unknown origin (Fig. 2). Hierarchical clustering was also performed individually for each of the six infectious diseases using the symptoms that were present in at least 10% of the records for each of the disease, to gain further disease-specific insights on symptom co-occurrence and its distribution (S1 Fig).

### ML-based models using symptoms for CoD prediction

To confirm if symptoms can be used to predict CoDs for each record, ML-based classification models were built individually for each of the six diseases. Each model was build using 80% of training data and the remaining as test data. Six ML algorithms viz., SVM, GBM, C5, ANN, kNN and CART were used to predict CoD. The prediction performances of these models were evaluated based on accuracy, kappa, recall/sensitivity, specificity, precision and F1 score (Table 2 and S2 Fig).

**Table 2:** Comparison of the prediction accuracy of ML-based algorithms for six diseases

ML algorithms	Pneumonia	Diarrhoeal diseases	Malaria	Meningitis/ encephalitis	Measles	Fever of unknown origin
**SVM**	0.90	0.97	0.91	0.80	0.92	0.87
**GBM**	0.90	0.97	0.92	0.80	0.92	0.79
**C5**	0.89	0.97	0.90	0.80	0.92	0.87
**ANN**	0.89	0.96	0.90	0.79	0.93	0.88
**kNN**	0.89	0.96	0.91	0.75	0.93	0.87
**CART**	0.87	0.96	0.83	0.75	0.92	0.81

Of 6 ML-based algorithms, SVM and GBM performed better than the other four algorithms (Table 2). Literature evidences also suggest that SVM models are superior for developing disease classification models [44, 45].

The SVM based prediction model performed best for diarrhoeal diseases and lowest for meningitis/encephalitis (Table 3). SVM models could classify pneumonia, diarrhoeal diseases, malaria, meningitis/encephalitis, measles and FOUO with 91, 95, 90, 83, 97 and 87% precision respectively using the associated symptom data (Table 3).

**Table 3:** Performance matrix of SVM models for six infectious diseases

Performance Measure	Pneumonia	Diarrhoeal diseases	Malaria	Meningitis/ encephalitis	Measles	Fever of unknown origin
**Accuracy**	0.90	0.97	0.91	0.80	0.92	0.87
**Kappa**	0.80	0.93	0.81	0.60	0.84	0.74
**Recall/Sensitivity**	0.88	0.98	0.92	0.76	0.88	0.87
**Specificity**	0.92	0.95	0.90	0.85	0.97	0.87
**Precision**	0.91	0.95	0.90	0.83	0.97	0.87
**F1 score**	0.90	0.97	0.91	0.79	0.92	0.87

**Fig. 3:**
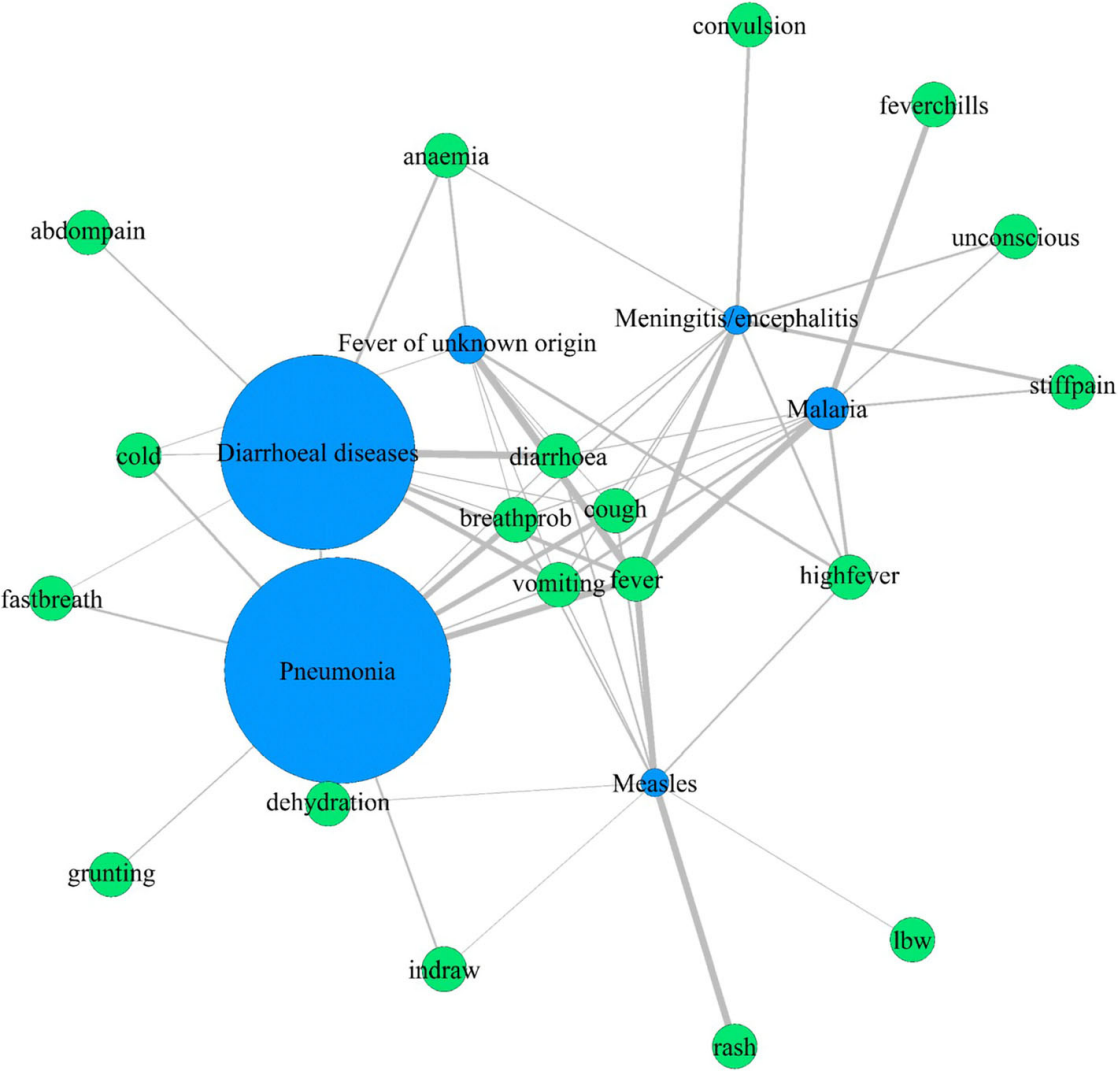
Disease-symptom network of top 10 features obtained from SVM model. Green nodes represent symptoms and blue nodes represent diseases. The size of disease node is proportional to number of records corresponding to the disease in the dataset. Edge represents association between disease and symptom and its width is proportional to percentage of records positive for the symptom. The network was created using *igraph* R package [48]

The 10 most relevant symptoms for CoD prediction for each of the SVM-based prediction models were also extracted (S3 Fig) and this data concurs well with WHO manual for disease diagnosis [41–43, 46, 47]. The co-occurrence of these top 10 features of each of the six diseases was visualised using disease-symptom network plot (Fig. 3). Of 35 symptoms used for the ML-based disease model generation, 19 symptoms were critical for classification of the six diseases and five symptoms viz., fever, diarrhoea, breathing problem (breathprob), cough and vomiting were associated with all the six infectious diseases (Fig. 3). Six symptoms viz., abdominal pain /distention (abdompain), convulsion, fever chills, grunting, low birth weight (lbw) and rash were identified as important predictors specifically for a single disease (Fig. 3). For eg., rash was identified as one of the top predictors specifically for measles while grunting was an important predictor only for pneumonia.

### SVM models for classifying diseases with overlapping symptoms

SVM models were built for classifying pairs of diseases that had several overlapping symptoms. The performance measures of SVM models for predicting CoD for pneumonia-diarrhoeal diseases, malaria- meningitis/encephalitis and malaria-fever of unknown origin are shown in Table 4 and the 10 most important features for disease prediction can be viewed in S4 Fig. SVM model for pneumonia- diarrhoeal diseases depicted highest accuracy (98%). SVM models for malaria – meningitis/encephalitis and malaria - fever of unknown origin were able to classify with 84 and 85% accuracy respectively.

**Table 4:** Performance matrix of SVM models for pairwise disease classification

Performance Measure	Pneumonia- Diarrhoeal diseases	Malaria - Meningitis /encephalitis	Malaria - Fever of unknown origin
**Accuracy**	0.98	0.84	0.85
**Kappa**	0.95	0.67	0.70
**Recall/Sensitivity**	0.98	0.81	0.87
**Specificity**	0.98	0.87	0.83
**Precision**	0.98	0.86	0.84
**F1 score**	0.98	0.83	0.85

## Discussion

Using ML-based algorithms, we could effectively predict CoD from the signs/symptoms captured by the VA tools. We have documented the ability of the symptoms to form disease-based clusters, in spite of being present in multiple diseases, suggesting that they can be effectively exploited as input variables to predict the corresponding CoDs.

Although all the ML algorithms (except CART) performed well for disease prediction, SVM models displayed consistently superior performance for all six diseases. In previous studies on disease prediction, SVMs using RBF kernel have found to be better performers than other ML-algorithms such as SVMs with linear or polynomial kernels, Random Forest and Decision Trees [44, 45].

Our study is, as far as we can determine, the first to systematically compare various ML-based algorithms applied to physician coded VAs. While there has been substantial debate if algorithms outperform physician coding, a recent randomized trial among 10,000 deaths showed the physician coding outperformed most currently available algorithms [16]. Moreover, the worldwide clinically accepted standard of medical diagnosis or of certification of the causes of deaths are by physicians. Our paper adds to the literature suggesting that ML-assisted algorithms may help to improve and standardize physician-based coding. This is especially relevant for childhood conditions, where the major reasons for death are few, and reasonably similar across African and Asian countries [49].

The strengths of the study were its large size, representative sampling of deaths in India and standard ways of coding of records by physicians. Moreover, the keywords used by physicians while variable, were amenable for binning into broader categories that permitted reducing the input feature space and application of ML-algorithms. Nonetheless the study has some limitations. Three important parameters that are missed in this study are the type, duration and intensity of the illness. Hence, symptoms such as dry or wet cough; cough for a week or a month; intense vomiting/diarrhoea over mild vomiting/diarrhoea cannot be distinguished. The study relies on the cognitive abilities of the respondents and in cases where the death has occurred in distant past, the recollection of the symptoms may not be perfect.

## Conclusions

For the foreseeable future, national verbal autopsy studies are critical to capture rural, home deaths until a time when deaths start to occur mostly in facilities that mandate medical certification. Under these circumstances, innovations to improve verbal autopsy methods are essential. ML-algorithms applied to physician-derived keywords offer a simple, practicable way to improve the classification of causes of death in children, and should be considered as one of the strategies for advances in verbal autopsy methodology.

## Supplementary Information

**Additional file 1.**

## Data Availability

The MDS dataset is the property of the Government of India and cannot be shared. Requests to access the MDS data need to be approved by the Registrar General of India- https://censusindia.gov.in/AboutUs/Contactus/Contactus.html.

## Abbreviations

ANN: Artificial neural network
CART: Classification and regression tree
CoD: Cause of death
GBM: Gradient boosting modelling
ICD: International classification of diseases
kNN: k-nearest neighbour
MDS: Million Death Study
ML: Machine learning
SVM: Support vector machine
VA: Verbal autopsy
WHO: World Health Organization
