# Supplementary material for: Comparison of machine learning algorithms applied to symptoms to determine infectious causes of death in children: national survey of 18,000 verbal autopsies in the Million Death Study in India
Source: BMC Public Health. 2021 Oct 4;21:1787. doi: 10.1186/s12889-021-11829-y (PMC8488544; doi:10.1186/s12889-021-11829-y)
Supplement: Supplementary file 1 — Additional file 1. [file 12889_2021_11829_MOESM1_ESM.docx]

**Supporting Information**

**Supplementary Appendix**

**S1 Appendix: Physicians keyword categorization for 35 symptom categories with inclusion and exclusion terms.**

| Symptom category | | Inclusion terms | Exclusion terms |
| --- | --- | --- | --- |
| Fever* | | fev OR high temp OR rise in temp | no fev OR typhoid fev OR brain fev OR fever of brain OR cerebral fev OR high grade fev OR high fev OR fever high grade OR severe fev OR chill OR rigor OR shiv |
|  | High-grade fever | high grade fev OR high fev OR fever high grade OR severe fev |  |
|  | Fever with chills | chill OR rigor OR shiv | no chill OR no rigor OR without chill OR without rigor OR no shiv |
|  | Night sweats | night AND sweat | no night sweat |
| Cold temperature | | cold body OR body cold OR become cold OR became cold OR cold extrem OR cold chest OR cold expos OR exposure to cold OR exposed to cold OR feeling cold OR hypotherm OR cool body OR decreased body temp OR winter OR cold weather | no cold exposure OR no exposure to cold OR not exposed to cold |
| Cold/coryza/rhinitis | | cold OR coryza OR rhin OR congestion OR congested chest OR running nose OR runny nose OR nasal discharge OR nose block OR watering from nose OR sneezing | no cold OR no coryza OR no rhin OR cold body OR body cold OR cold water OR become cold OR became cold OR cold exposure OR exposure to cold OR exposed to cold OR cold climate OR cold weather OR cold extremities OR cold chest OR no congestion OR feeling cold OR hot and cold |
| Breathing problems* | | breathing prob OR breathing troub OR difficult bre OR difficulty bre OR breathing dif OR difficulty in bre OR laboured bre OR breathles OR brethles OR dyspn OR dysn OR dysponea OR respiratory prob OR respiration prob OR respiratory diff OR respiration diff OR difficulty in resp OR shortness OR sob OR breathing dis OR chest prob | no breath OR no difficult bre OR no difficulty bre OR no difficulty in bre OR not breathles OR not brethles OR no dyspn OR no dysn OR no respiratory prob OR no respiration prob OR no respiratory diff OR no respiration diff OR no difficulty in resp OR no shortness OR no sob OR no associated sob |
|  | Chest indrawing | chest in OR indraw OR in-draw OR in draw OR chest retract OR rib retract OR intercostal reces OR intercostal retract OR chest withd OR abdominal bre | no chest in OR no indraw OR no in-draw OR no in draw OR no chest retract OR no rib retract OR chest infect |
|  | Fast/rapid breathing | fast bre OR breathing fast OR fast resp OR rapid bre OR rapid resp OR tachypn OR tachyop OR tachyap OR increased resp OR increased bre OR hypervent OR fast chest OR high resp OR breathing rate inc OR respiratory rate inc OR raised rr OR increased rr | no fast bre OR no breathing fast OR no fast resp OR not fast bre OR not breathing fast OR not fast resp OR no hypervent |
|  | Grunting | grunt OR stridor OR noisy bre OR noisy resp | no grunt OR no stridor |
|  | Wheezing | wheez OR whistl | no wheez OR no whistl |
|  | Respiratory distress | respiratory distr OR resp dist | no respiratory dist OR no resp dist |
| Cough* | | cough OR expectorat | no cough |
|  | Blood in sputum | (blood AND sput) OR hemopt OR haemopt | no blood OR no hemopt OR no haemopt |
| Chest pain | | chest pain OR pain in chest OR chestpain OR painful resp OR chest discom OR pain in the chest OR chest tight | no chest pain OR no pain in chest OR no chestpain |
| Turned blue | | blu OR cyan OR cyn | no blu OR not blu OR no cyan OR not cyan OR no cyn |
| Convulsion | | convu OR fit OR seiz | no convu OR no fit OR benefit OR benifit OR benfit OR no seiz |
| Unconsciousness | | unconsc OR unconc OR unconsious OR loss of con OR lost con OR coma OR drowsy OR loc OR faint | local OR lock OR megalo OR bloc |
| Stiffness/body pain | | stif OR myalgia OR body pain OR joint pain OR headache OR head ache OR bodyache OR body ache OR muscle pain OR pain in limb OR neck rigid | no stif OR not stif OR no neck stif OR no body stif OR no myalgia OR no body pain OR no joint pain OR no headache OR no head ache OR no bodyache OR no body ache OR no muscle pain |
| Poor feeding | | poor feed OR difficulty feed OR difficulty in feed OR no feed OR not feed OR no breast feed OR loss of ap OR appetite loss OR decrease ap OR decreased ap OR lost ap OR reduced ap OR poor ap OR lack of ap OR anorex OR stopped breast OR poor breast OR stopped eat OR stopped feed OR stopped taking f OR not take f OR not taking f OR reduced food OR unable to eat OR unable to feed OR unable to suck OR poor suck OR not suck OR not able to suck OR no suck OR stop suck OR stopped suck OR stop feed OR decreased food OR decreased feed OR refus OR ((reduced OR poor OR stopped) AND oral intake) | no difficulty feed OR no loss of ap OR no appetite loss OR no anorex OR not anorex OR refused admission |
| Low birth weight | | lbw OR low birth weight OR low birth wt OR prem OR preterm OR pre term OR pre-term OR ((small OR low) AND (baby OR birth OR date)) | no lbw OR no low birth weight OR no low birth wt OR no prem OR not prem |
| Loss of weight | | ((loss OR lost) AND (weight OR wt)) OR malnour OR malnut OR undernour OR undernut OR became thin OR became very thin OR became lean | no weight OR no loss |
| Anaemia | | anem OR anaem OR aneam OR pallor OR pale OR became weak OR became very weak OR become weak OR become very weak OR weak since birth OR weak at birth OR weak from birth OR letharg | no anem OR no anaem OR no aneam OR not anem OR not anaem OR not aneam |
| Diarrhoea* | | diar OR dair OR dirrh OR loose OR ((watery OR loos OR lose OR liquid) AND (stool OR mot)) OR dysent OR gastro | no diar OR no dair OR no dirrh OR no loose OR no lose OR no watery OR no dysent OR no liquid stool OR rice water OR no gastro |
|  | Blood in stools | blood AND stool | no blood |
|  | Dehydration | dehydr OR low urin OR less urin OR no urin OR decrease urin OR decrease in urin OR decreased urin OR reduced urin OR oligur OR anur OR yellow urin OR yellowish urin OR urine yellow OR thirst OR sunken eye OR shrunken eye | no dehydr |
| Abdominal pain/ distention | | ((abd OR stomach) AND (pain OR disten OR discom OR swell OR swoll OR prob)) OR indigest OR bloat OR (cramps AND (abd OR intest)) | no abd OR no pain OR no stomach OR no disten OR no indigest OR no bloat OR no cramp |
| Jaundice | | jaund OR (yellow AND (eye OR skin OR discol)) OR icterus | no jaund OR no yellow OR no icterus |
| Swelling | | swell OR edema OR swoll | no swell OR swollen abd OR abdomen swoll OR abdominal swell OR swelling of abd |
| Rash | | rash OR spot OR pox OR chechak OR variola OR varicella OR eruption OR papu OR pappu | no rash OR no spot OR no chechak OR no variola OR no varicella OR no eruption OR no papu OR no pappu |
| Vomiting | | vom OR hematem OR naus | no vom OR no hematem OR no naus |
| Typhoid | | typhoid | no typhoid |
| Cholera | | cholera OR (rice AND water) | no cholera OR no rice |
| Abscess/pus | | abscess OR pus | no abscess OR no pus OR push |
| Delirium | | delir OR deler OR altered sens OR confus | no delir OR no deler |

**S2 Appendix: Count of death records (n = 13,216) for ages 1-59 months with physician keywords reflecting 35 symptom categories for six infectious causes of death.**

|  | | Cause of death | | | | | |
| --- | --- | --- | --- | --- | --- | --- | --- |
| Symptom category | | Pneumonia  (n = 5733) | Diarrhoeal diseases  (n = 4897) | Malaria  (n = 860) | Fever of unknown origin  (n = 754) | Meningitis/ encephalitis  (n = 490) | Measles  (n = 482) |
| Fever* | | 4793 | 2356 | 843 | 754 | 407 | 408 |
|  | High-grade fever | 952 | 282 | 304 | 256 | 130 | 88 |
|  | Fever with chills | 607 | 167 | 634 | 49 | 55 | 20 |
|  | Night sweats | 5 | 1 | 3 | 0 | 0 | 0 |
| Cold temperature | | 113 | 35 | 3 | 1 | 2 | 0 |
| Cold/coryza/rhinitis | | 1681 | 141 | 44 | 18 | 19 | 36 |
| Breathing problems* | | 4238 | 325 | 68 | 25 | 74 | 75 |
|  | Chest indrawing | 1221 | 28 | 3 | 1 | 5 | 7 |
|  | Fast/rapid breathing | 1433 | 67 | 6 | 1 | 14 | 17 |
|  | Grunting | 489 | 8 | 2 | 1 | 5 | 2 |
|  | Wheezing | 410 | 8 | 3 | 0 | 1 | 2 |
|  | Respiratory distress | 234 | 12 | 1 | 2 | 0 | 6 |
| Cough* | | 3671 | 286 | 70 | 22 | 46 | 93 |
|  | Blood in sputum | 18 | 3 | 0 | 0 | 1 | 0 |
| Chest pain | | 181 | 8 | 5 | 0 | 2 | 0 |
| Turned blue | | 54 | 12 | 2 | 2 | 1 | 0 |
| Convulsion | | 156 | 81 | 86 | 16 | 188 | 13 |
| Unconsciousness | | 216 | 192 | 129 | 25 | 106 | 24 |
| Stiffness/body pain | | 134 | 106 | 178 | 20 | 218 | 15 |
| Poor feeding | | 586 | 401 | 137 | 79 | 34 | 62 |
| Low birth weight | | 424 | 255 | 11 | 45 | 23 | 9 |
| Loss of weight | | 170 | 262 | 44 | 23 | 10 | 22 |
| Anaemia | | 971 | 1623 | 149 | 196 | 61 | 84 |
| Diarrhoea* | | 438 | 4701 | 63 | 19 | 38 | 76 |
|  | Blood in stools | 8 | 310 | 2 | 0 | 1 | 6 |
|  | Dehydration | 24 | 1109 | 21 | 2 | 4 | 7 |
| Abdominal pain/distention | | 155 | 706 | 27 | 17 | 19 | 22 |
| Jaundice | | 142 | 134 | 79 | 11 | 11 | 5 |
| Swelling | | 45 | 42 | 3 | 6 | 11 | 6 |
| Rash | | 35 | 46 | 4 | 4 | 13 | 434 |
| Vomiting | | 669 | 2985 | 296 | 47 | 54 | 41 |
| Typhoid | | 8 | 157 | 3 | 2 | 1 | 2 |
| Cholera | | 1 | 174 | 0 | 0 | 0 | 0 |
| Abscess/pus | | 5 | 4 | 1 | 0 | 2 | 27 |
| Delirium | | 5 | 2 | 42 | 3 | 4 | 1 |

* For the categories “fever”, “breathing problems”, “cough”, and “diarrhoea”, records in the subcategories are included into the overall category counts. For example, record counts for the “fever” category include records keywords categorized as “fever”, “high-grade fever”, “fever with chills”, and/or “night sweats”.

**Supplementary Figures**


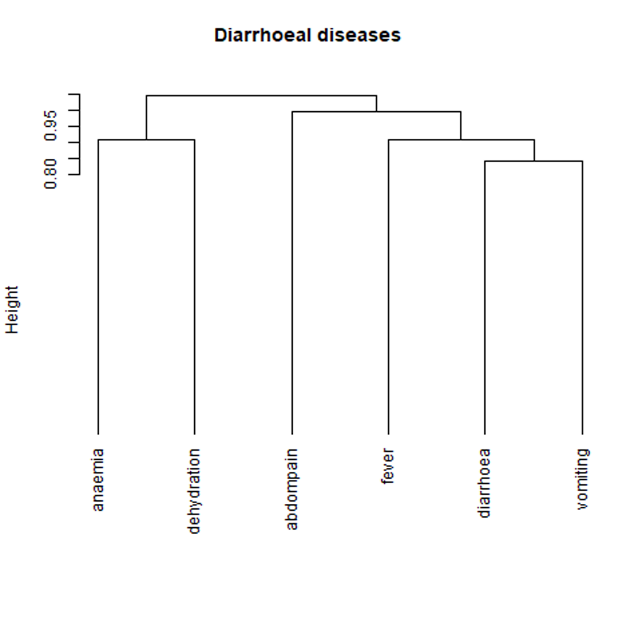

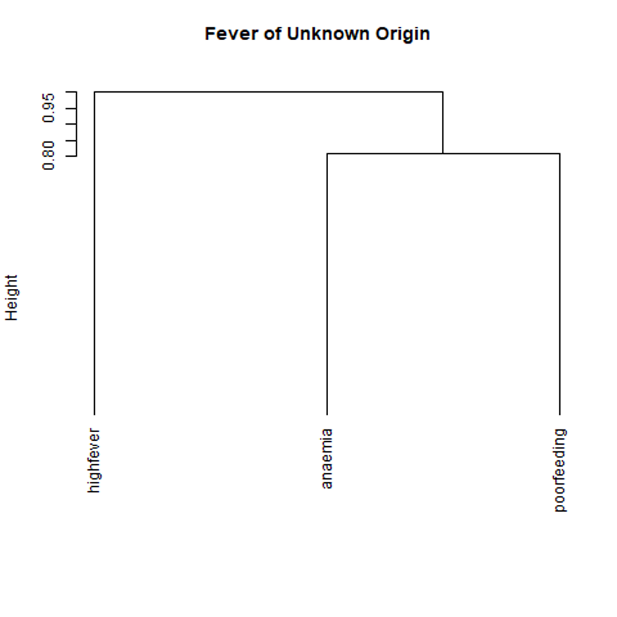


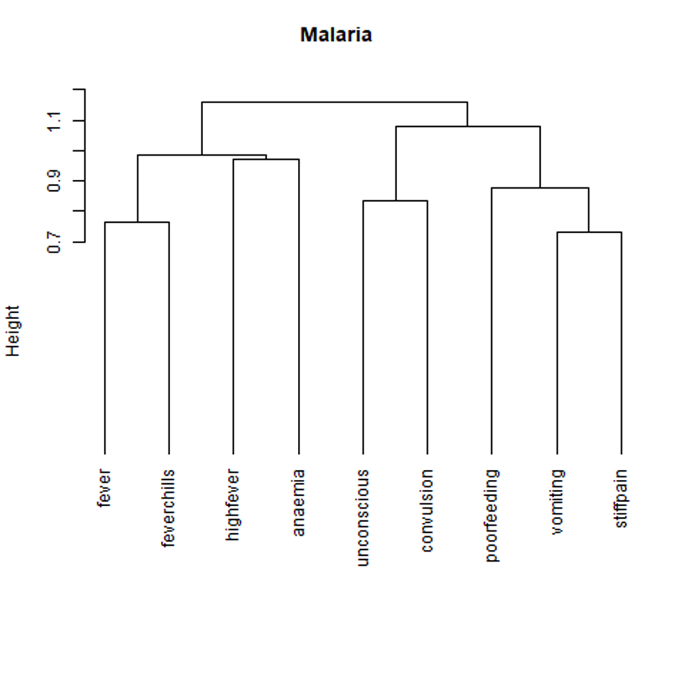

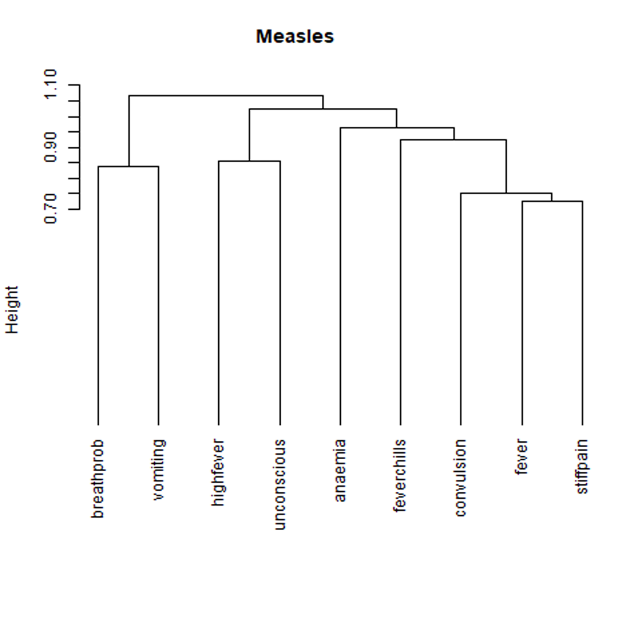


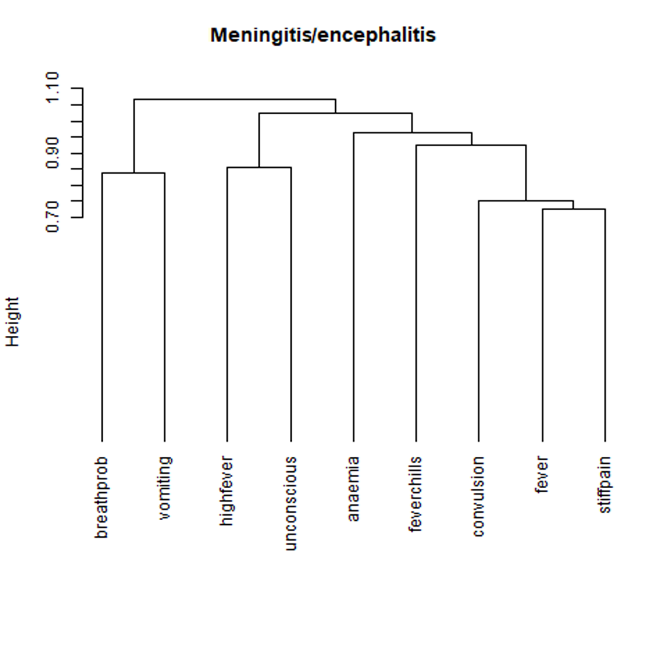

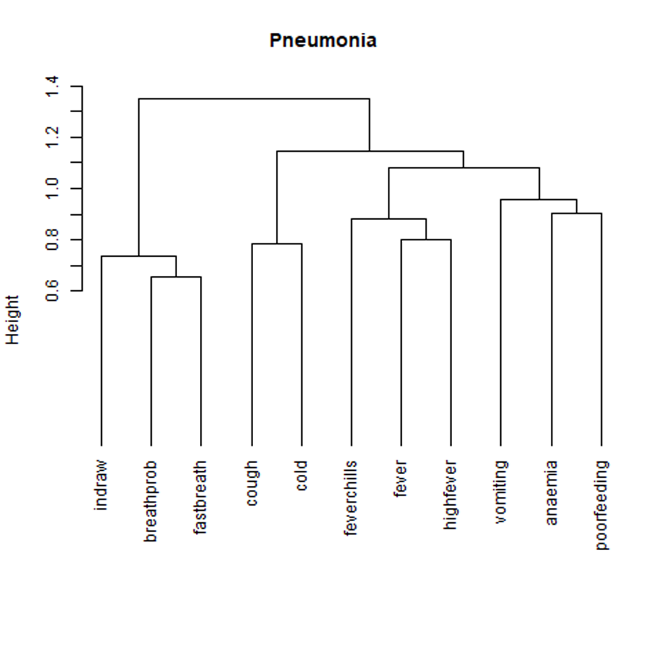


**S1 Fig. Tree-based clustering of the symptoms that were present in at least 10% of the records for each of the six diseases. The vertical axis represents the distance between clusters.**

| 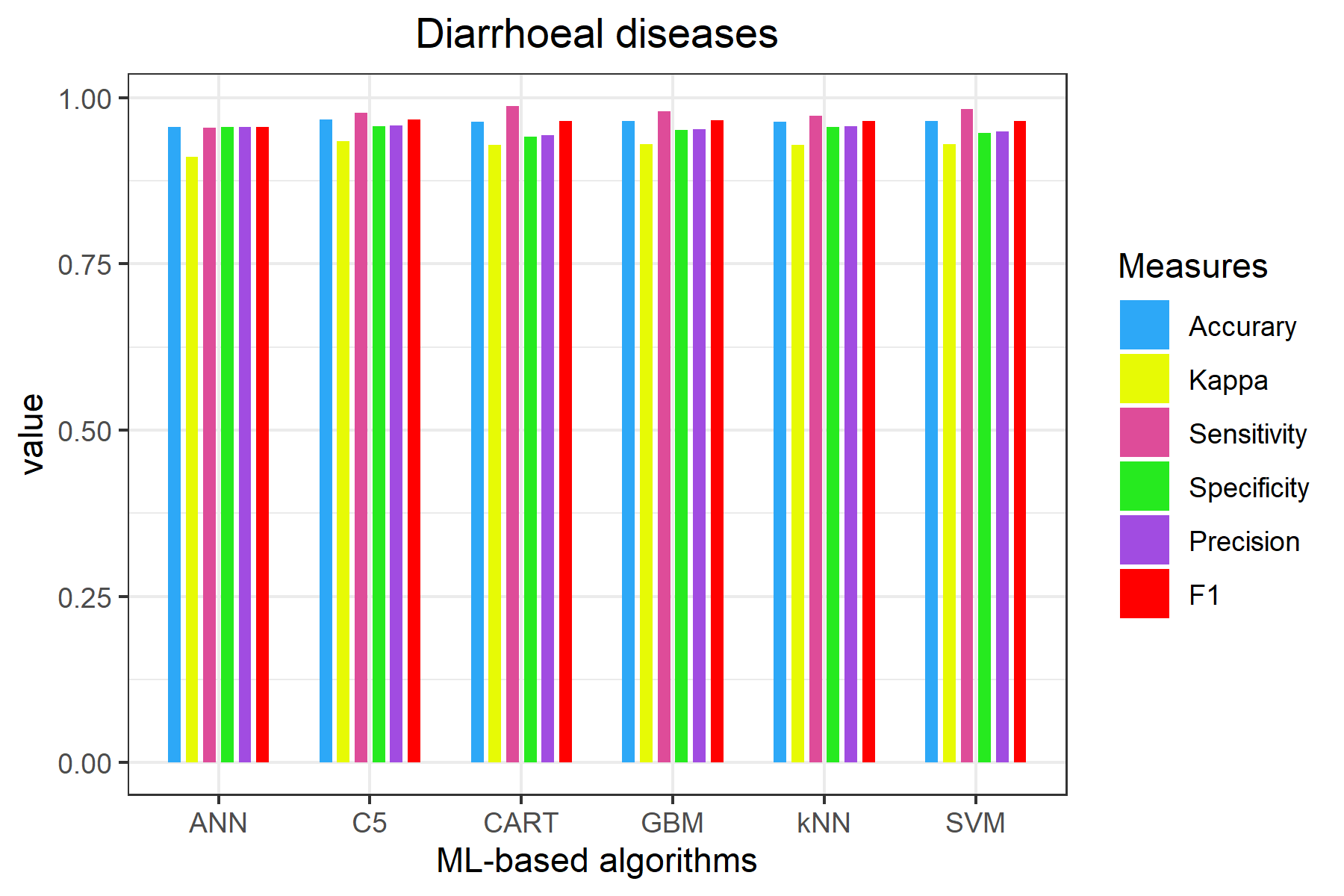 | 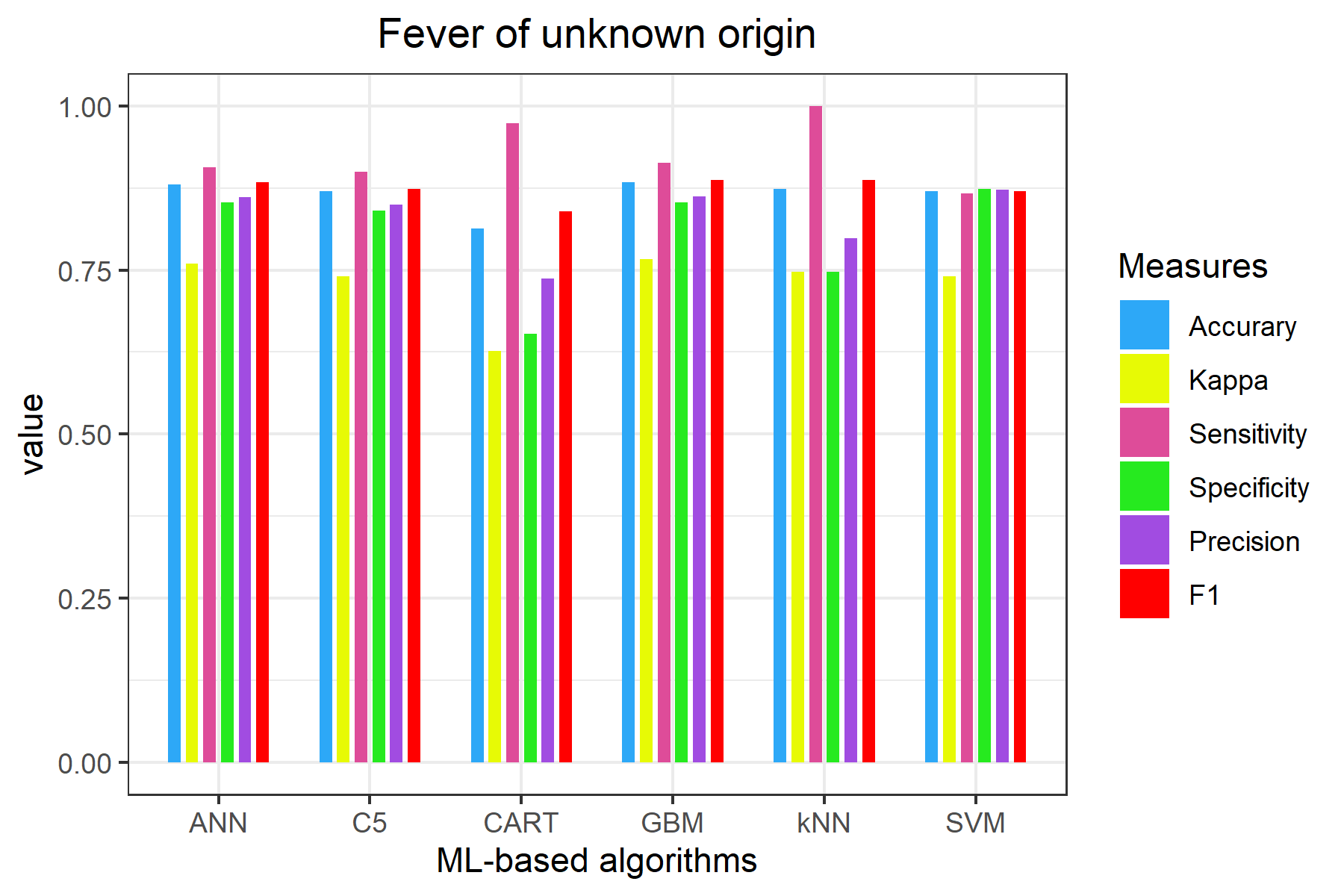 |
| --- | --- |
| 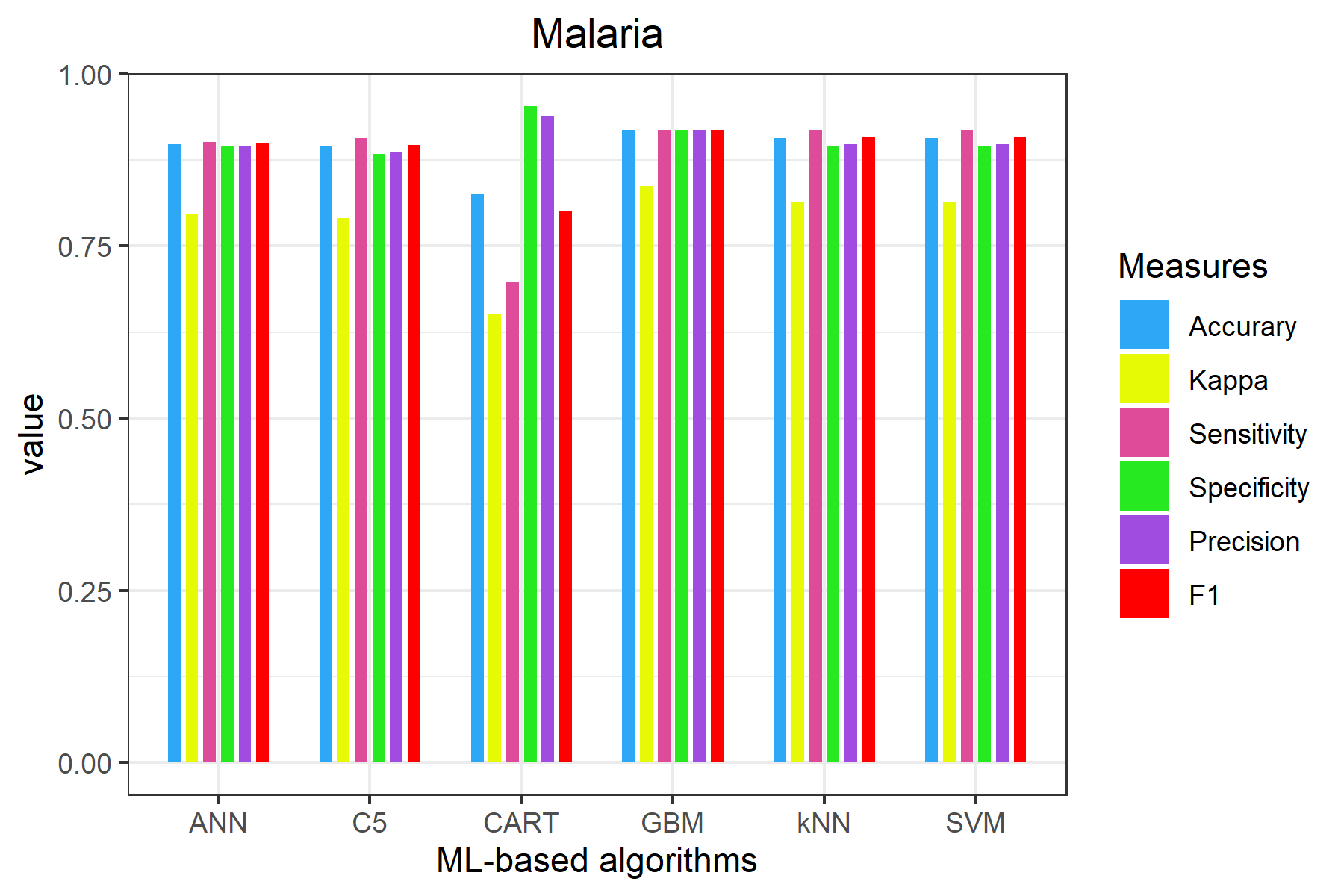 | 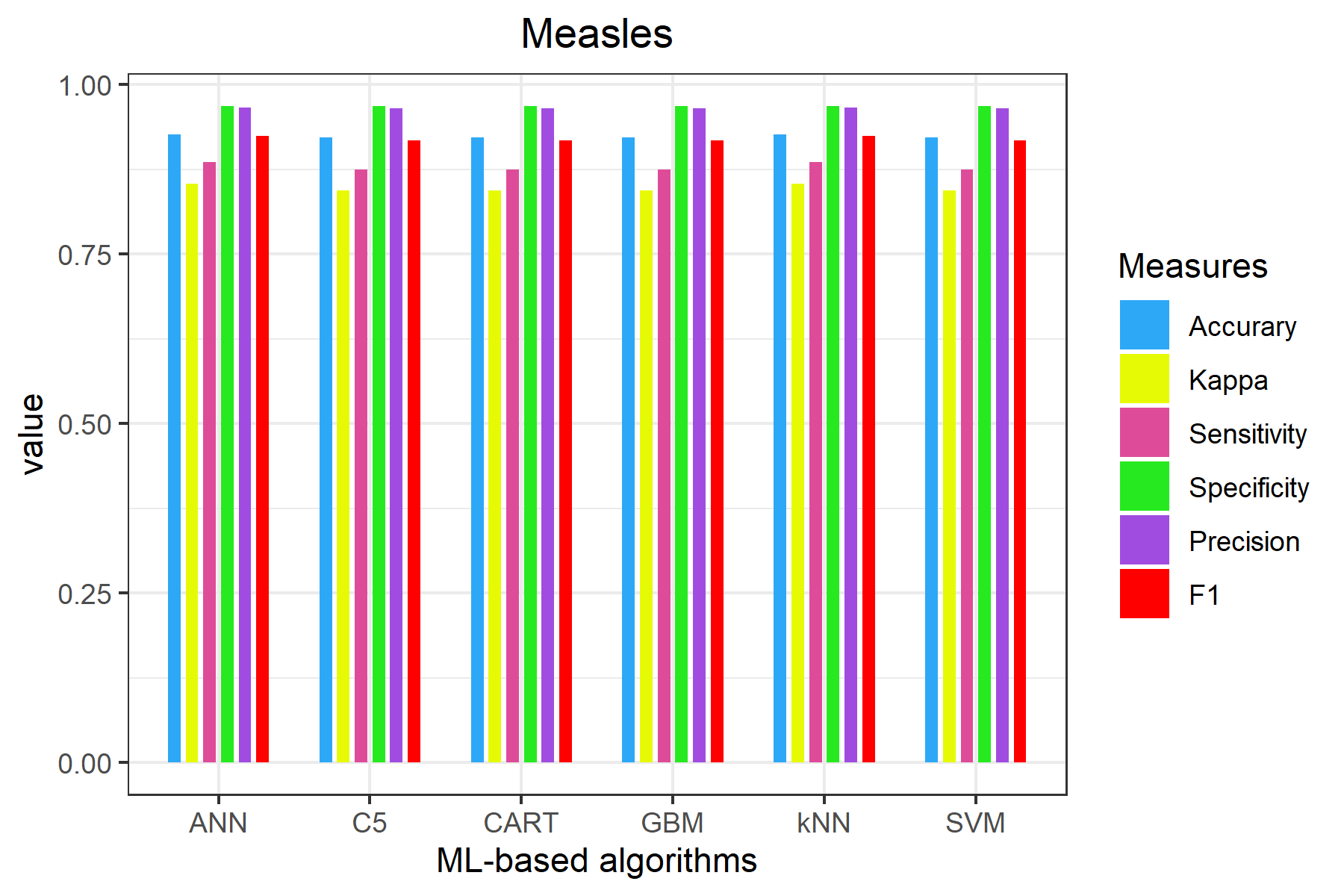 |
| 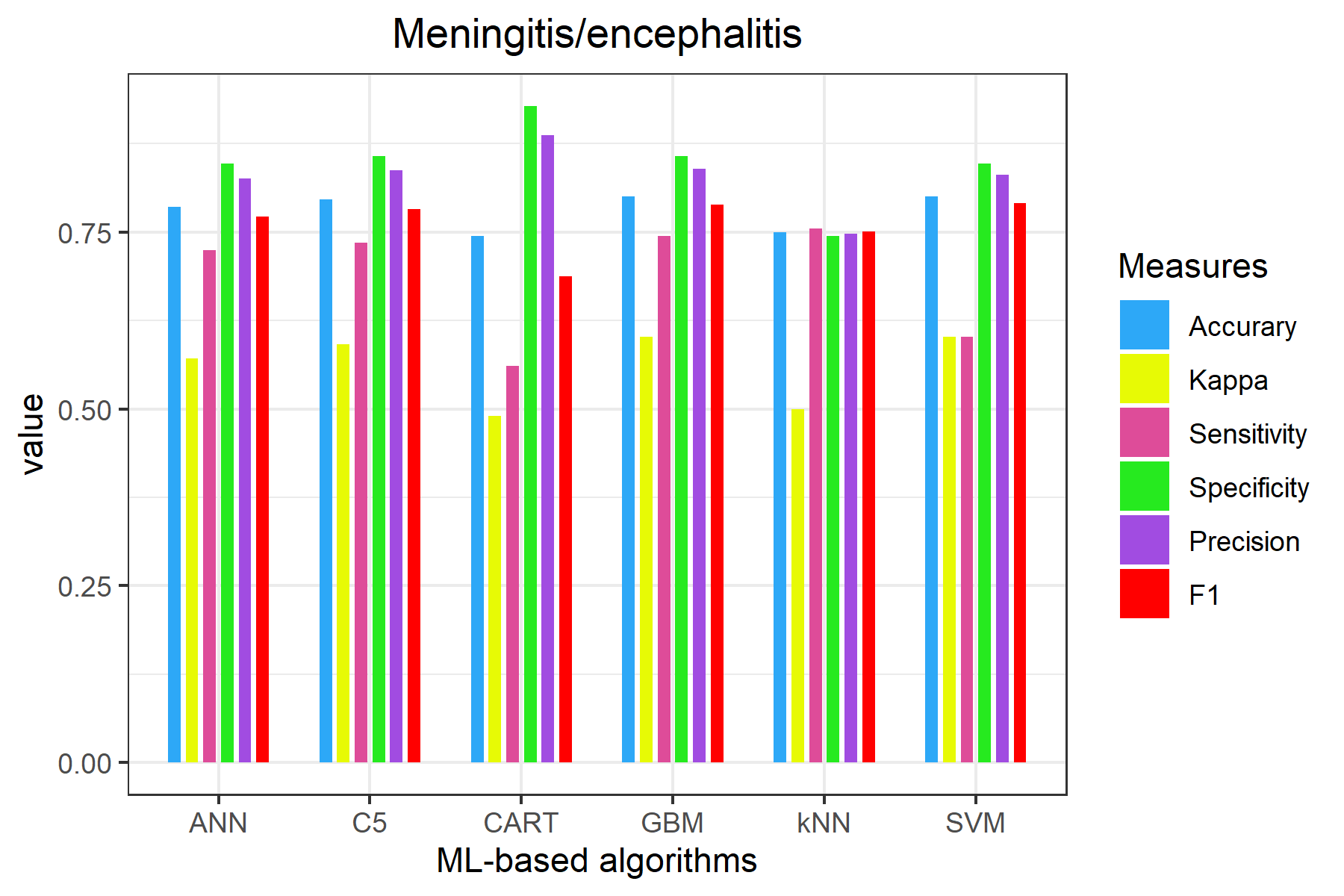 | 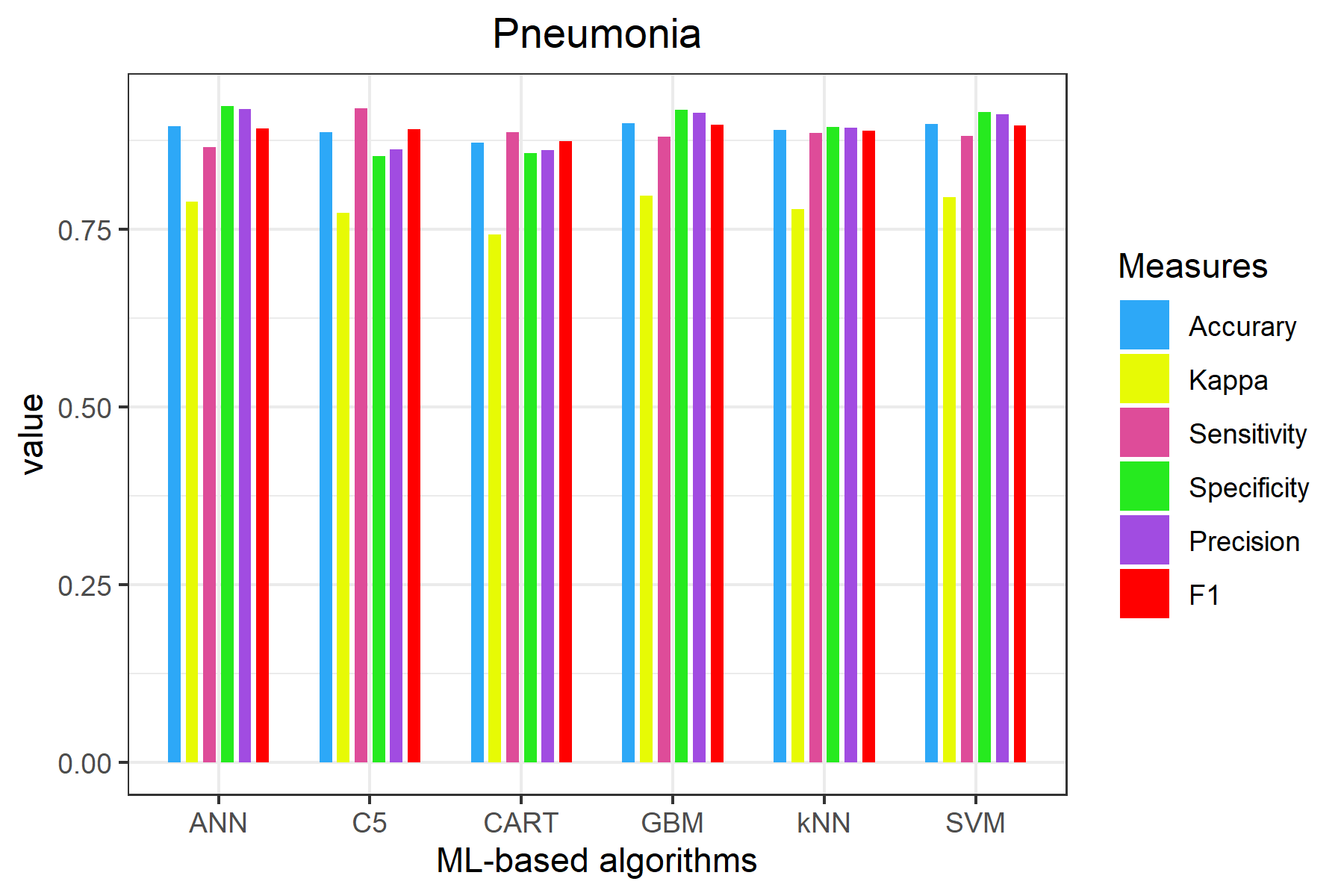 |

**S2 Fig. Prediction performance of ML models for six diseases. The x-axis represents the ML-based algorithms and y-axis represents the values of performance measures for ML models. The colored bars represent the accuracy, kappa, sensitivity, specificity, precision and F1 values of the ML models.**

| **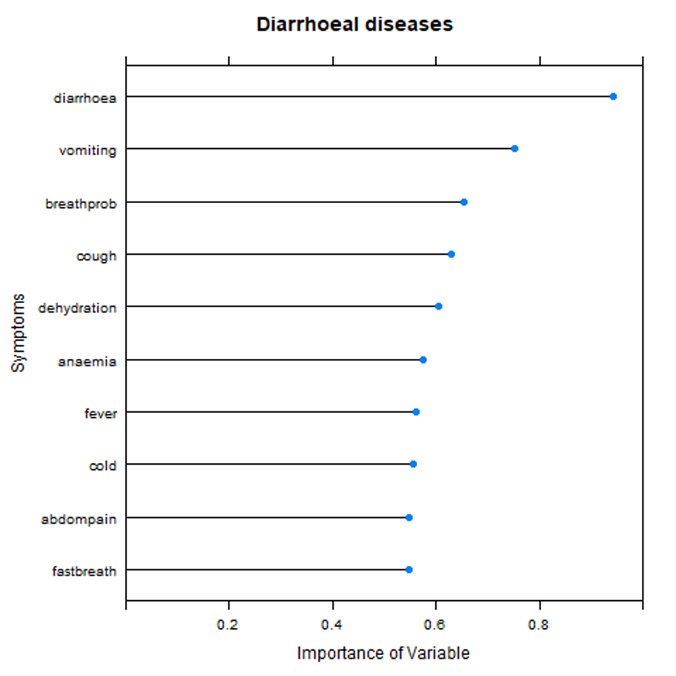** | **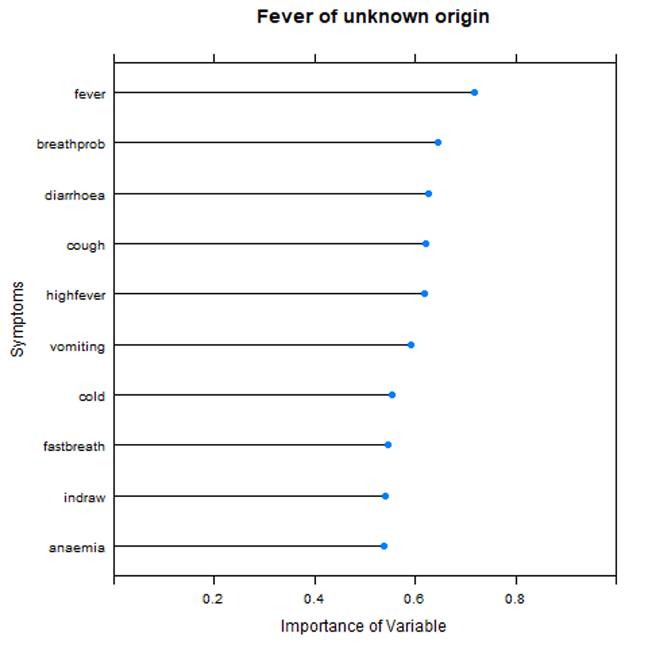** |
| --- | --- |
| **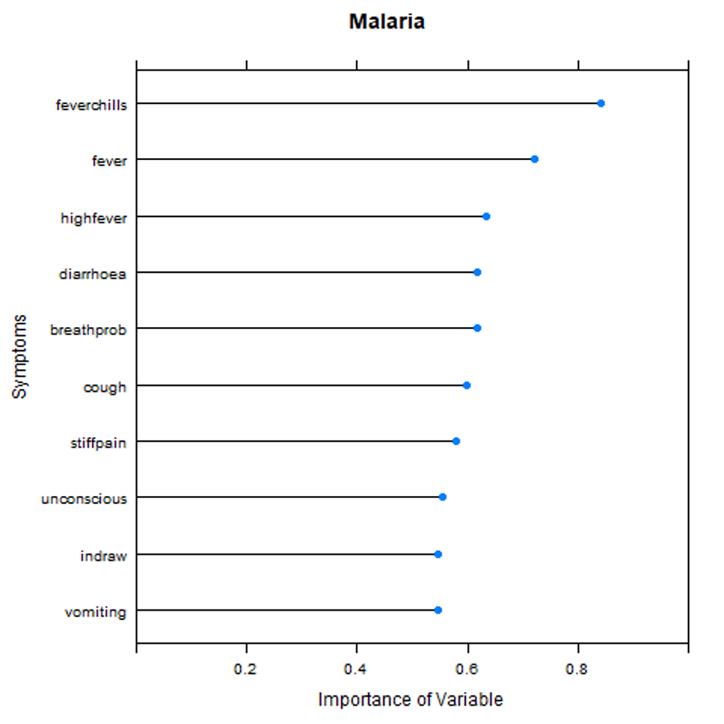** | **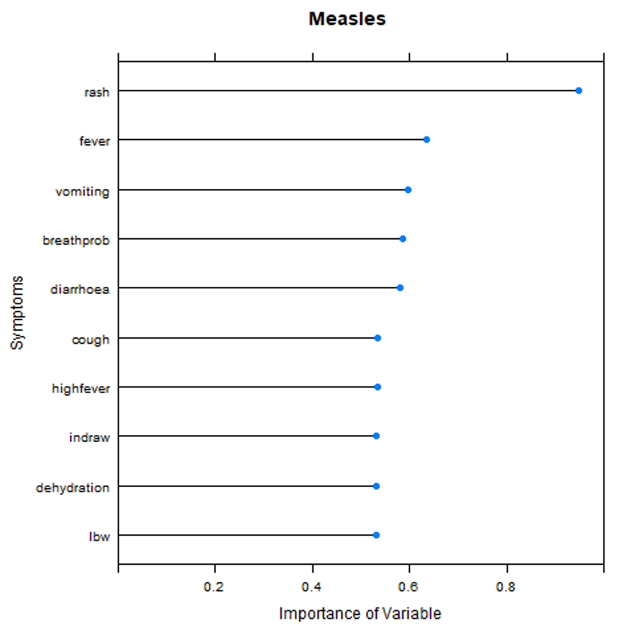** |
| **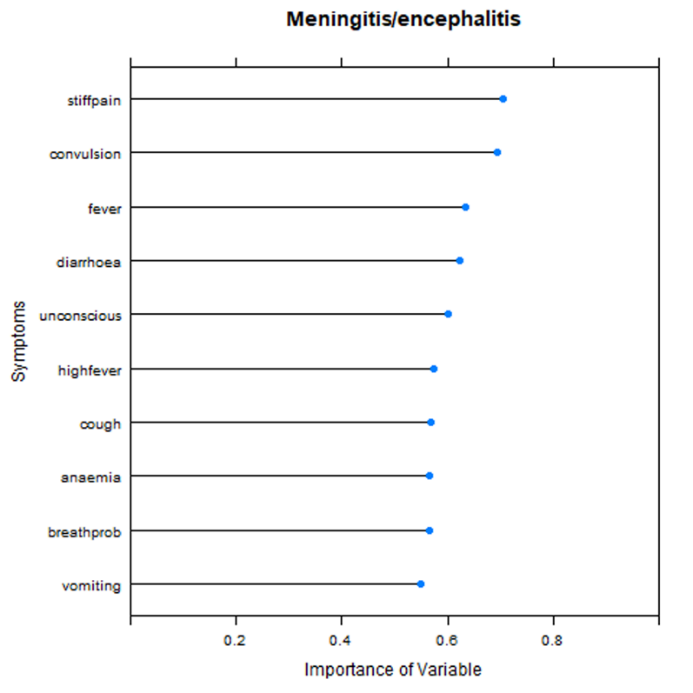** | **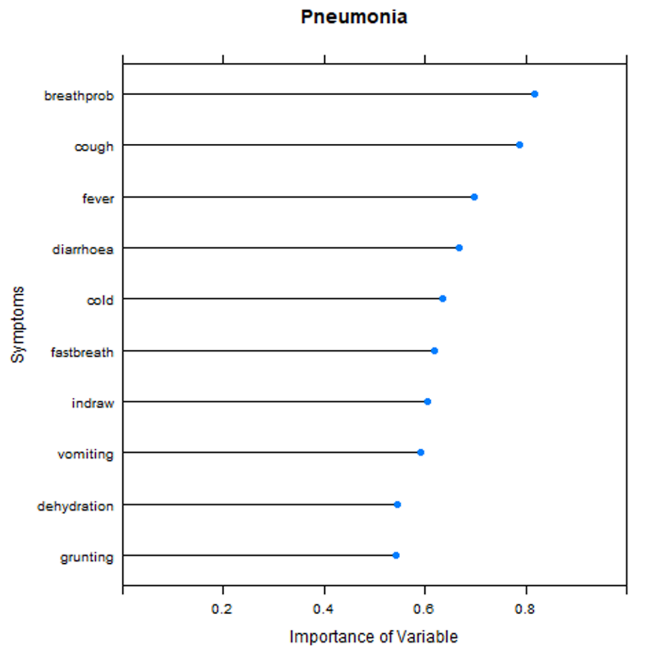** |

**S3 Fig. Top 10 features selected for prediction of the six diseases in SVM model. The x-axis represents the importance of variable in the prediction model and y-axis represents the symptoms.**

**
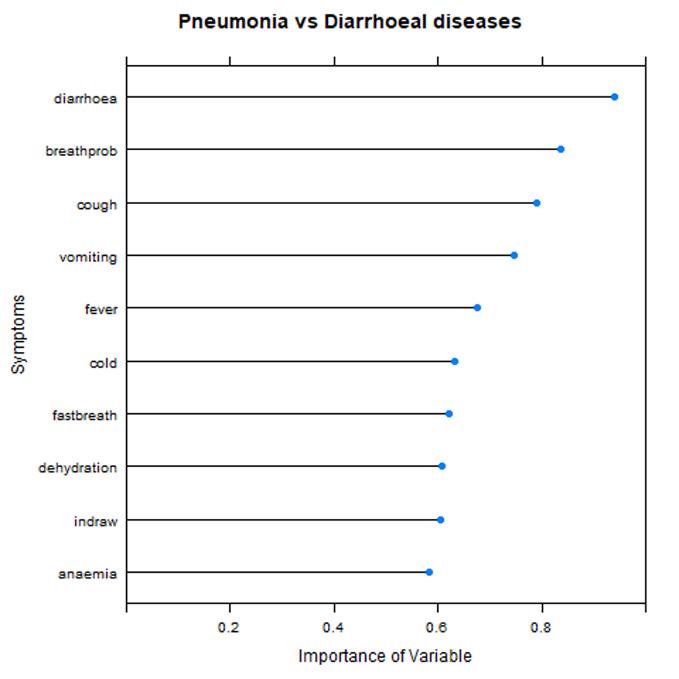

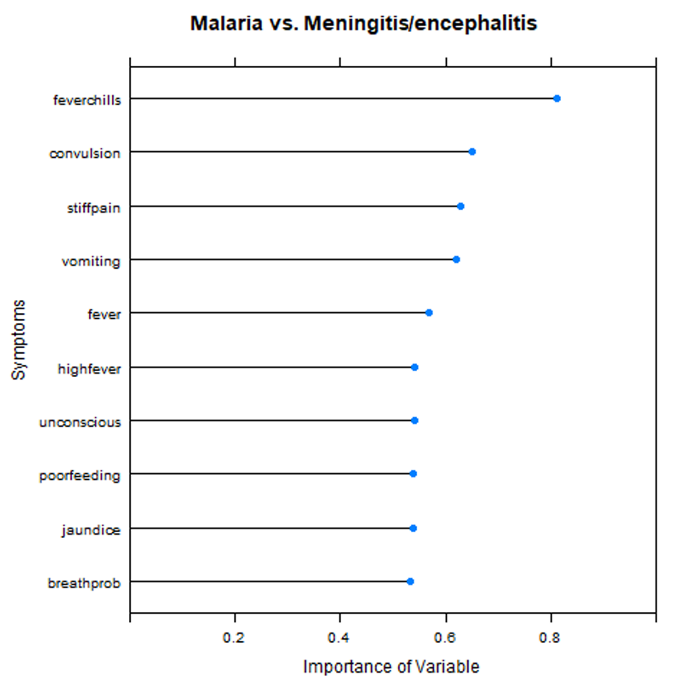

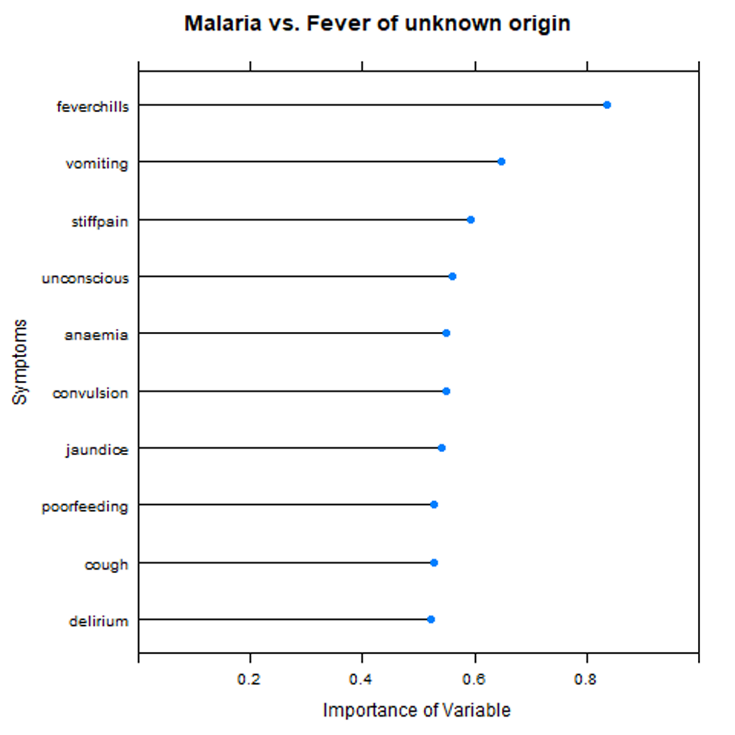
**

**S4 Fig. Top 10 features selected for classification of the CoDs studied pairwise using SVM. The x-axis represents the importance of variable and y-axis represents the symptoms.**
